# Development of an Alternative Low-Cost Larval Diet for Mass Rearing of *Aedes aegypti* Mosquitoes

**DOI:** 10.1155/2020/1053818

**Published:** 2020-11-24

**Authors:** Umesha Senevirathna, Lahiru Udayanga, G. A. S. M. Ganehiarachchi, Menaka Hapugoda, Tharaka Ranathunge, Nilmini Silva Gunawardene

**Affiliations:** ^1^Department of Zoology and Environmental Management, Faculty of Science, University of Kelaniya, Sri Lanka; ^2^Department of Biosystems Engineering, Faculty of Agriculture and Plantation Management, Wayamba University, Sri Lanka; ^3^Molecular Medicine Unit, Faculty of Medicine, University of Kelaniya, Sri Lanka

## Abstract

**Background:**

*Aedes aegypti* is a major vector of arboviruses that may be controlled on an area-wide basis, using novel approaches such as Sterile Insect Technique (SIT) and Incompatible Insect Technique (IIT). Larval diet is a critical factor to be considered in mass rearing of *Aedes* mosquitoes for SIT and IIT programs. Therefore, the current study is aimed at evaluating the effects of two novel diets developed from dry fish powder on the growth and development of immature stages and adult fitness-related characteristics of *Ae. aegypti* in Sri Lanka.

**Method:**

Three batches of the first instar *Ae. aegypti* larva, each containing 250 larvae, were exposed to three different larval diets as standard dry fish powder (D_1_), dry fish powder meal and brewer's yeast (D_2_), and International Atomic Energy Agency- (IAEA-) recommended diet (D_3_), separately. Morphometric and developmental parameters of the 4^th^ instar larvae, pupae, and adult mosquitoes reared under different dietary treatments were measured. The entire experimental setup was replicated thrice. A General Linear Model (GLM) in the form of two-way ANOVA was used for the statistical analysis.

**Results:**

Significant diet-based variations were observed in the head length, head width, thoracic length, thoracic width, abdominal length, abdominal width, and total length (*F*_2,87_ > 4.811; *P* < 0.05) of *Ae. aegypti* larvae. The highest pupation success and the larval size were observed from the larvae fed the D_2_ diet, while the lowest was reported from D_1_. All adult morphometric parameters of adult male and female *Ae. aegypti* mosquitoes also denoted significant dietary variations, reporting the best-sized adults from the D_2_ diet (*F*_2,87_ > 3.54; *P* < 0.05). Further, significantly higher fecundity and male longevity were also shown by the adult *Ae. aegypti* (*F*_2,6_ > 7.897; *P* < 0.01) mosquitoes reared under diet D_2_.

**Conclusion:**

Based on all the growth and developmental parameters, the D_2_ diet tends to perform similar to the IAEA-recommended diet in mass rearing of *Ae. aegypti* mosquitoes, while being more inexpensive. Therefore, larval diet D_2_ could be suggested as the ideal diet for mass rearing of *Ae. aegypti* for IIT and SIT-based vector control in Sri Lanka.

## 1. Background

Dengue is a mosquito-borne viral disease that has challenged more than 129 countries in different parts of the world in recent years. About 390 million dengue viral infections occur every year, and virtually, 3.9 billion people live in dengue-endemic countries [1]. Approximately, 1.8 billion people (more than 70%) at risk for dengue infection reside within the Western Pacific and Southeast Asia regions, contributing to 75% of the global disease burden of dengue [2]. According to the World Health Organization (WHO), the global incidence of dengue has denoted a 30-fold increment within the last five decades [1].

Sri Lanka has been affected by regular epidemics of Dengue Fever (DF) and Dengue Haemorrhagic Fever (DHF) for over two decades. Dengue viral (DENV) infections have been endemic in Sri Lanka since the mid-1960s. DF was serologically confirmed on the island in 1962, while the first epidemic of dengue in Sri Lanka was experienced in the 1980s [3]. Regular epidemics of dengue have been reported from Sri Lanka since 2009, with an average case burden of 35,000–45,000 per year. However, due to recent changes in virus serotype(s), Sri Lanka is facing a dramatic increase in the incidence of dengue leading to an alarming situation at present [4]. The highest number of dengue cases was reported in 2017 as 186,101 with over 350 deaths [5]. Approximately, 20,000 to 25,000 deaths due to dengue are reported at the global level annually [1]. Meanwhile, a total of 105,049 dengue cases were reported from Sri Lanka in 2019.

In Sri Lanka, *Aedes aegypti* acts as the primary vector of dengue, while *Ae. albopictus* contributes as the secondary vector. Due to the absence of an effective drug or vaccine for dengue, patient management and vector control are considered the most effective strategies to control dengue in the country [4]. Up to now, different conventional methods are being used for dengue control, with emphasis on vector control. Environmental management, chemical-based vector control methods, community participation, and biological control could be identified as the current vector control strategies used for the suppression of *Aedes* vectors in Sri Lanka [6, 7].

Significant adverse impacts on nontarget populations, development of resistance to insecticides, higher financial costs, and relative incompetence of chemical vector control approaches have encouraged the government entities to consider alternative methods for vector control in Sri Lanka [8]. Therefore, novel and innovative approaches such as the use of genetically modified mosquitoes, Sterile Insect Technique (SIT), and Incompatible Insect Technique (IIT) are being considered to effectively control dengue outbreaks in Sri Lanka [9].

SIT is a species-specific and eco-friendly method used since 1977 to control insect pests [10]. This technique involves the release of a large number of irradiated sterile male insects into the environment, allowing them to compete with wild fertile males for mating with wild female insects. On the other hand, IIT is a strategy where some symbiotic bacteria are used for the production of antipathogen effector molecules. Often, *Wolbachia*, a Gram-negative naturally transmitted endosymbiotic bacterium, is used in IIT to induce feminization in males, parthenogenesis, and cytoplasmic incompatibility and to decrease adult life span of males [11]. As both IIT and SIT rely upon the release of sterile male mosquitoes in large numbers, mass rearing of *Aedes* vectors is one of the principal requirements in the process [12]. It is a challenging task as the production of mosquitoes in sufficient numbers and of adequate quality is crucial for the success of IIT and SIT approaches. Further, since the integration of SIT into an Area-Wide Integrated Pest Management (AW-IPM) program competes economically with other control techniques, the production of *Aedes* mosquitoes must be timely and cost-effective [9].

In the mass rearing phase, mainly the rearing conditions and larval diet quality have a direct and often irreversible effect on adult traits [13]. According to Timmermann and Briegel [14], a larval diet should provide a wide range of nutrients to avoid the risk of deficiencies that could negatively affect both the rearing productivity and fitness of the mosquitoes produced. When selecting a diet for mass rearing purposes, the components in the diet also play an important role. Since high in nutrient ingredients, such as wheat germ, soy flour, ground beef, and chicken eggs, are locally available at inexpensive prices, development of an optimum larval diet for mass rearing of *Aedes* mosquitoes with readily available ingredients would be more profitable [15].

In contrast, the International Atomic Energy Agency (IAEA) has recommended a specifically designed diet to be used for mass rearing, seeking to provide adequate nutritional components to ensure optimum growth rates and performance in *Aedes* mosquitoes. IAEA diet has been widely utilized for mass rearing of *Ae. aegypti* in many developing countries including Sri Lanka. However, the IAEA diet should be either imported or locally prepared, both of which are costly [16, 17]. Therefore, many countries are pursuing to develop more economical alternative larval diets, which are readily available and provide the same nutritional properties to *Ae. aegypti* larvae [9]. Therefore, the current study was conducted to evaluate the performance of two locally developed larval diets against the IAEA-recommended diet to be used for mass rearing of *Ae. aegypti* in Sri Lanka, while ensuring optimum morphometric development and adult sexual competitiveness, under laboratory settings.

## 2. Methods

### 2.1. Acquiring of *Ae. aegypti* Larvae

*Ae. aegypti* eggs (obtained from F_5_ generation) were obtained from the indoor insectary of the Molecular Medicine Unit (MMU), Faculty of Medicine, University of Kelaniya (7°1′41.99^″^N and 79°55′38.99^″^E). Collected eggs were transferred to 1 L plastic trays with deoxygenated water and kept for 8 hours for hatching in the Arthropod Containment Facility at the MMU. Hatched larvae were counted and transferred into properly labeled plastic trays (40 cm × 30 cm × 5 cm) containing 2 L of distilled water [18]. For the purpose of quality control and maintenance of uniform size of the larvae, a density of 1000 larvae per tray was maintained.

Initially, larvae were fed 1.5 mL of IAEA-recommended larval diet. Then, the larval diet was added once a day to a tray according to the following regime: day 1, 1.5 mL; day 2, 1.55 mL; day 3, 1.6 mL; day 4, 1.65 mL; and day 5, 1.7 mL [17]. Once larvae developed into the pupal stage (there will be a mixture of larvae and pupae in the tray), pupae were counted and transferred to 500 mL plastic cups containing distilled water, using a Pasteur pipette.

### 2.2. Rearing of the Adult *Ae. aegypti*

Pupal rearing cups were kept inside the adult mosquito rearing cages (30 × 30 × 30 cm), until the emergence of adults. After the emergence of adult mosquitoes, they were counted and transferred into an adult rearing cage. Adults were held in properly labeled cages (30 cm × 30 cm × 30 cm) with a density of seven hundred adult mosquitoes per cage, while maintaining a 1 : 1 male : female ratio. Adult mosquitoes were fed with a 10% sucrose solution. Sugar solutions were replaced every two days to avoid any fungal growth inside the cage. Mosquitoes were reared in an environment with a temperature of 26–27°C and 78–80% RH under a 12-hour light and 12-hour dark cycle [16].

After 3 days since emergence, adult mosquitoes were fed with cattle originated blood. The sucrose solution was removed from the adult cages 12-24 hours prior to feeding with a blood meal [19]. For feeding, 5 mL of cattle blood was poured into the Hemotek membrane feeder (PS-6 System, Discovery Workshops, Accrington, UK) that maintained the temperature of the blood at 37 ± 1°C. The feeder was then placed on top of the adult cage allowing the female mosquitoes to feed for around 1-2 hours. Subsequently, 48 hours after the blood feeding, egg collection was done by placing an egg collection cup (250 mL) containing distilled water (10 mL), cotton, and egg-laying filter paper, inside the cage. Egg papers were removed from the cages and left to dry under the standard conditions for 24 hours. The first instar larvae that emerged from above eggs were taken for the experiment by following the standard hatching procedure [19].

### 2.3. Formulation of Larval Diets

In the current study, efficacy of two different larval diets was compared with that of the IAEA-recommended larval diet. Stock solution of the first diet (D_1_) was formulated by dissolving 25 g of dry fish powder meal (available at the Peliyagoda fish market, Peliyagoda, Sri Lanka) in 100 mL of distilled water. The dry fish powder was comprised of fish, shellfish, and mollusks, with crude protein (19% wt/wt), crude fat (5% wt/wt), and ash (4% wt/wt), as specified by the manufacturer. Meanwhile, the second larval diet (D_2_) was prepared by dissolving 21.5 g of dry fish powder meal and 3.5 g brewer's yeast in 100 mL of distilled water. Stock slurry of the third larval diet (D_3_) was prepared according to the recommended compositions of the International Atomic Energy Agency as the control by dissolving 50% tuna meal (12.5 g) (T.C. Union Agrotech, Thailand), 36% bovine liver powder (9.0 g) (MP Biomedicals, Santa Ana, CA), and 14% brewer's yeast (3.5 g) (Sigma Aldrich Inc., St. Louis, MO) in 100 mL of distilled water [13]. The homogenized stock slurries of the three diets were stored at -20°C to prevent the degradation of diet components and microbial proliferation [18]. The initial concentrations of the prepared stock slurries were considered 100% throughout the study.

### 2.4. Larval Feeding Experiment

Three batches of 250 first instar *Ae. aegypti* larvae (L_1_) were counted and transferred into three separately labeled (D_1_, D_2_, and D_3_) larval rearing trays (25 × 25 × 7 cm) containing 500 mL of deionized water. Each tray was treated with the relevant larval diet (among three larval diets) in appropriate volumes, as mentioned above. Fecal matter and debris in the larval trays were removed daily using Pasture pipettes, in order to maintain satisfactory water quality levels for larval development. The pupae and adults emerging from three treatments were maintained under standard conditions as described above (Section 2.1).

### 2.5. Determination of the Life History Parameters

Randomly selected larval samples of the fourth instar larvae (*n* = 10) and pupae (*n* = 10) were collected from each diet treatment into the Eppendorf tubes with 80% ethanol. In addition, randomly selected adult males (*n* = 10) and females (*n* = 10) that emerged from each diet treatment were captured from the cages using a mouth aspirator and put into collection vials, separately after labeling them. Subsequently, they were killed and preserved by chilling immediately in the refrigerator at 4°C. All of the above-described experiments were repeated three times to maintain the accuracy of the findings.

Morphometric parameters of the preserved fourth instar *Ae. aegypti* larvae, namely, head length, head width, thoracic length, thoracic width, abdominal length, abdominal width, and total length, were measured in a straight position using a digital USB camera fixed on a stereomicroscope and OPTIKA version 2.12 image processing software under magnification (10x), as described by Gunathilaka et al. [17]. In addition, the mean pupation success of *Ae. aegypti* larvae was calculated separately for each larval diet as the percentage of larvae pupated from the total number of introduced larvae. In pupae, the cephalothoracic length and width were measured in the straight position under magnification (10x). The mean adult success rates were determined as the percentage of adults that emerged in relation to the total number of pupae introduced [17].

The right wings of each preserved male and female mosquitoes from separate diet treatments were dissected under a dissecting microscope. Dissected wings and thorax with abdomen were mounted on glass slides, separately. The standardized wing length from the distal alula to the end of the radius excluding the fringe scale and the width of the wing at the greatest breadth excluding fringe were measured under magnification (10x) [20, 21]. In addition, the thoracic length (from the base of the neck to the base of the abdomen), thoracic width (from dorsum to the coxae), abdominal length (from the base of the tip), and abdominal width (at greatest width) were also measured using the digital USB Optica camera mounted on the stereomicroscope under magnification (10x). The number of eggs laid by 100 blood-fed females (2 days after blood feeding) was recorded for the three larval diet treatments, separately. In addition, the collected eggs were hatched, and the fertility rates were calculated as the percentage of the number of hatched L_1_ larvae to the number of eggs laid [18]. Further, the number of days required to achieve the 50% mortality of the male mosquitoes that emerged from different larval diets was reported as male longevity [22].

After 24 hours since emergence, 100 pupae of one sex were placed in a petri dish (6 cm in diameter and 1.5 cm in height) that opens into a glass tube (20 cm in height and 7 cm in diameter) and placed in an acrylic cage (30 × 30 × 30 cm). Flight ability was calculated as the percentage of adults that were able to exit from the tube into the cage over an observation period of 48 hours.

### 2.6. Statistical Analysis

All the data was entered into Microsoft Excel worksheets, adhering to quality control procedures. IBM SPSS Statistics (version 23 copyright by IBM Corporation) was used for data analysis. The effect of different larval diets on morphometric parameters of larvae, pupae, and adults was investigated by using the General Linear Model (GLM) followed by Tukey's HSD for mean separation at a 5% level of significance. Further, significance in the variations of mean pupation success, adult success, fecundity, fertility, male longevity, and adult flight ability over different larval diets was also analyzed using GLM.

## 3. Results

### 3.1. Morphometric Parameters of the 4^th^ Instar Larvae and Mean Pupation Success

The mean growth parameters (head length, head width, thoracic length, thoracic width, abdominal length, and abdominal width) of *Ae. aegypti* 4^th^ instar larvae fed three different larval diets are indicated in Table 1. As the results denote, there was a significant diet-based variation in the head length (*F*_2,87_ = 14.491; *P* < 0.001), head width (*F*_2,87_ = 13.907; *P* < 0.001), thoracic length (*F*_2,87_ = 22.829; *P* < 0.001), thoracic width (*F*_2,87_ = 23.273; *P* < 0.001), abdominal length (*F*_2,87_ = 14.814; *P* < 0.001), abdominal width (*F*_2,87_ = 4.811; *P* = 0.01), and total length (*F*_2,87_ = 7.279; *P* = 0.001) at 95% level of confidence.

As indicated by the results, the highest values for all the studied growth parameters, namely, head length (0.57 ± 0.01 mm), head width (0.60 ± 0.01 mm), thoracic length (0.88 ± 0.01 mm), thoracic width (1.04 ± 0.01 mm), abdominal length (3.59 ± 0.01 mm), abdominal width (0.70 ± 0.01 mm), and total length (4.98 ± 0.04 mm), were observed from the 4^th^ instar larvae fed diet 2 (D_2_), while the lowest values of all the parameters were observed from the larvae fed diet 1 (D_1_), as shown in Table 1. Interestingly, head length, head width, thoracic length, thoracic width, abdominal length, and abdominal width of the 4^th^ instar larvae of *Ae. aegypti* fed larval diets 2 (D_2_) and 3 (IAEA) belonged to the same cluster, as denoted by the post hoc analysis of GLM (Table 1).

Meanwhile, mean pupation success of *Ae. aegypti* larvae fed different larval diets also denoted significant variations (*F*_2,6_ = 20.544; *P* = 0.001) at a 95% level of confidence (Table 1). Furthermore, the highest mean pupation success (89.0 ± 2.3%) was observed from the pupae that emerged from the larvae fed D_2_ diet, while the lowest was reported from larval diet D_1_ as 78.0 ± 1.5% (Table 1). However, the mean pupation success rates of larval diets D_2_ and D_3_ did not show any significant difference.

### 3.2. Impact of the Type of Larval Diet on Pupal Developmental Parameters

**Figure 1:**
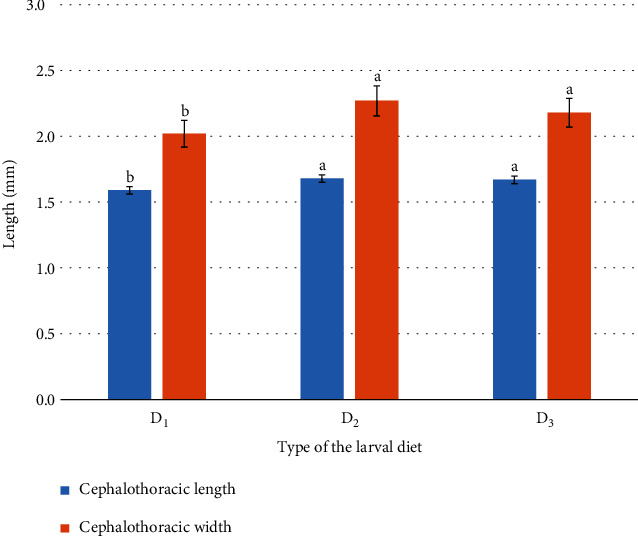
Mean cephalothoracic length and width of the *Ae. aegypti* pupae formed from larvae treated with different larval diets. Note: different letters over columns denote significant differences (*P* < 0.05) at a 95% level of confidence based on the General Linear Model followed by Tukey's pairwise comparison.

Both cephalothoracic length (*F*_2,87_ = 7.803; *P* = 0.001) and width (*F*_2,87_ = 34.181; *P* < 0.001) of the *Ae. aegypti* pupae, formed from larvae treated with different larval diets, denoted significant differences (*P* < 0.05) at 95% level of confidence (Figure 1). The highest cephalothoracic length and width of 1.68 ± 0.01 mm and 2.27 ± 0.02 mm, respectively, were observed from the pupae raised from the D_2_ larval diet. On the other hand, the D_1_ diet accounted for the lowest (Figure 1).

**Figure 2:**
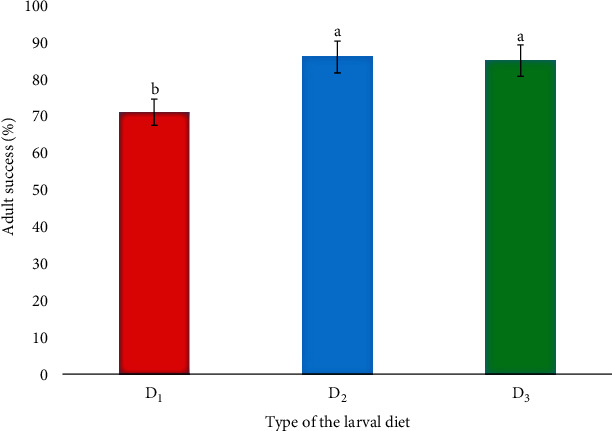
Mean adult success rates of the *Ae. aegypti* larvae reared under different larval diets. Note: different letters over columns denote significant differences (*P* < 0.05) at a 95% level of confidence based on the General Linear Model followed by Tukey's pairwise comparison.

As depicted in Figure 2, the mean adult success rates of *Ae. aegypti* pupae reared under different larval diets also denoted a significant variation with the diet (*F*_2,6_ = 11.19; *P* = 0.0066) at a 95% level of confidence. The highest mean adult success rate of 86.0 ± 1.0% was shown from the larvae fed the D_2_ diet, while the lowest mean adult success rate (71.0 ± 3.0%) was observed from the larvae fed the D_1_ diet.

### 3.3. Impact of Larval Diet on Adult Developmental Parameters

Mean morphometric parameters of adult male and female mosquitoes formed from larvae reared under different larval diets are tabulated in Table 2, along with the results of the GLM. All adult morphometric parameters of adult male and female *Ae. aegypti* mosquitoes varied significantly with different larval diets (*F*_2,87_ > 3.54; *P* < 0.05 at 95% level of confidence). Comparatively, larger adult males of *Ae. aegypti* mosquitoes emerged from the D_2_ larval diet treatment, with the highest wing length (5.19 ± 0.03 mm), wing width (1.15 ± 0.02 mm), thoracic length (1.79 ± 0.01 mm), thoracic width (1.38 ± 0.02 mm), abdominal length (4.27 ± 0.04 mm), and abdominal width (0.82 ± 0.04 mm). On the other hand, the smallest male mosquitoes were observed from diet treatment 1 (D_1_) as shown in Table 2.

Similar to males, the highest wing length (5.27 ± 0.04 mm), highest wing width (1.35 ± 0.02 mm), highest thoracic length (1.92 ± 0.02 mm), highest thoracic width (1.45 ± 0.02 mm), highest abdominal length (4.25 ± 0.05 mm), and highest abdominal width (0.96 ± 0.03 mm) were observed from the adult females that emerged from the D_2_ diet treatment. On the contrary, the lowest growth parameters were observed from the adult females that emerged from diet treatment D_1_, except for wing width (Table 2).

### 3.4. Impact of the Type of Larval Diet on Behavior and Biology of *Ae. aegypti*

Fecundity (egg production per 100 blood-fed females) was significantly affected by different larval diets (*F*_2,6_ = 8.294; *P* = 0.014 at 95% level of confidence) provided during the larval stage based on the statistics of GLM (Table 3). The highest egg production (1453.4 ± 23.5) was observed from the females formed from larvae reared under diet 2 (D_2_), while the lowest egg production (1364.2 ± 12.8) was observed from the females of the D_1_ diet. However, the hatching rate/fertility was not significantly influenced by different larval diets given at the larval stages of *Ae. aegypti* (*F*_2,6_ = 4.397; *P* = 0.057) at a 95% confidence level (Table 3). However, the fertility rates of eggs produced by female mosquitoes of larval diets D_2_ (98.0 ± 0.3%) and D_3_ (97.5 ± 0.5%) remained relatively higher, while larval diet D_1_ reported the lowest hatching rate.

At the 95% level of confidence, there was a significant variation in the survival time (longevity) of the male adult mosquitoes (*F*_2,6_ = 7.897; *P* = 0.016) as shown in Table 3. The longest survival time (18.2 ± 0.4 days) was observed from the males that emerged from the D_2_ larval diet treatment, while the lowest survival time (16.0 ± 0.7 days) was observed from the adults that emerged from the D_1_ larval diet treatment. Further, even though the flight ability of adult mosquitoes did not show any diet-based variations (*F*_2,6_ = 21.427; *P* = 0.074), the adults fed diet 2 (D_2_) and IAEA diet (D_3_) denoted relatively higher flight abilities (>98.2 ± 0.2%) as indicated in Table 3.

## 4. Discussion

Success of novel biological control strategies such as SIT and IIT programs depends upon mass production of healthy and vigorous sterile insects to be released into target areas. For this, a balanced larval diet, which can ensure high survivorship and fast and homogeneous larval development, is desired. The use of a nutritious, yet cost-effective larval diet, is a key factor, which can influence the above aspects in mass rearing of *Aedes* vectors. Conditions faced during the larval stage of mosquitoes, such as water temperature, water depth of the container, and food quality and quantity, could influence the mosquito development and population size regulation [23, 24]. The larval diet of *Ae. aegypti* could limit their larval growth rate, survival period, and size [25–27]. Therefore, all the environmental conditions in the containers (except for the larval diet level) were uniformly maintained in this study to specifically investigate the effects of studied larval diets on different morphological and behavioral parameters of *Ae. aegypti*.

In the current study, *Aedes* larvae showed a significant difference in their head length, head width, thoracic length, and thoracic width due to different diet treatments. Only the abdominal length, abdominal width, and the total length did not show any significant difference with the diet treatment. In all these parameters, the highest growth level was observed from the larvae treated with the experimental diet D_2_ and IAEA-recommended diet, while the least growth was observed in D_1_. This reveals that there is a significant effect of adding yeast to the larval diet mixture to enhance the growth of *Ae. aegypti* larvae. Yeast has less protein and higher content of carbohydrates. Therefore, *Ae. aegypti* larvae can store and utilize their energetic components efficiently [28].

The pupation and adult success rates remain high, when all nutrients are abundant [29]. Thus, results of the current study evidence that both D_2_ and IAEA diets comprise of all the nutritional components (sugar and other digestible carbohydrates) required for larval development. The carbohydrate amount consumed in the larval stages is directly associated with pupation and adult emergence [30, 31]. Therefore, the significantly lower pupation and adult success rates shown in D_1_ diet may be due to the limited sugar availability, resulting in a developmental delay. Meanwhile, body size is more important for females as it influences a variety of factors including fecundity [32], level of dispersion, host attack rate [33], and blood feeding frequency [34]. Nutrient reserves of adults (mainly glycogen and triglycerides) obtained during the larval stage could extend the longevity of adult mosquitoes [35, 36].

Carbohydrates and lipids also influence the flight ability of adults. Results of the present study indicate that experimental diet D_2_ and IAEA-recommended diets are containing higher levels of carbohydrates and lipids, as both longevity and flight ability were significantly similar. Similarly higher longevity of males has been observed by Bond et al. [9], when using a Laboratory Rodent Diet (LRD) as a larval diet. Prolonged survival of male mosquitoes is a critical requirement for the success of IIT and SIT programs [37]. In the current study, experimental diet D_2_ performed better than the IAEA and D_1_ diets, in terms of adult male longevity.

Larval nutrient reserves (protein, lipid, and glycogen) are also important for egg production and the endocrine regulation of egg development in *Ae. aegypti* [38]. High levels of glycogen and protein can induce ovarian ecdysteroid production in females and inhibit juvenile hormone biosynthesis by the corpora allata, resulting in enhanced vitellogenesis and egg production [38]. On the other hand, few studies have reported that lipid reserves influence pupal commitment and the endocrine regulation of egg development in autogenous and anautogenous female mosquitoes [26, 38]. The current study reported a significantly higher fecundity and fertility from the adult mosquitoes reared under the D_2_ diet, suggesting that the newly formulated diet is performing well.

The IAEA diet, which is being used as a standard larval diet in many countries, includes tuna meal, bovine liver powder (BLP), and brewer's yeast, which are rich in proteins, vitamins, and fatty acids [15, 39]. However, local production of it is costly and challenging due to practical difficulties concerning the availability of the bovine liver powder component [40]. The evaluated experimental diets were comprised of accessible ingredients, namely, dry fish powder obtained from the fish market and yeast, which are readily available as an animal feed at a lower cost. Regardless of the absence of BLP and tuna meal, the experimental diets include discarded scales, skeletons of fish, and shellfish such as crustacean and mollusk, which are also rich in proteins [41].

Based on the performance of the experimental D_2_ diet, in terms of larval, pupal, and adult life developmental parameters and functional and behavioral parameters, it is clear that the D_2_ diet is an economic substitute for the IAEA diet. The IAEA-recommended diet has been estimated to cost approximately USD 64.26 per kilogram [15], while the D_2_ experimental diet costs only USD 2.32. Therefore, the present study reveals that the experimental D_2_ diet has a similar nutritional value to the IAEA diet ensuring the optimal development of *Ae. aegypti* larvae and production efficiency, making it a better larval supplement to be used in mass rearing of *Ae. aegypti*.

## 5. Conclusion

The findings of the current study revealed significant diet-based variations in *Ae. aegypti* larvae, in terms of all the morphometric parameters at a 95% level of confidence. The significantly largest larvae were produced from the D_2_ experimental diet, which also denoted the highest pupation success rate. Further, the cephalothoracic length and width also denoted significant differences among the dietary treatments, suggesting that the D_2_ diet results in the highest quality pupae in terms of morphometry and pupation success. All adult morphometric parameters of adult male and female *Ae. aegypti* mosquitoes also varied significantly with different larval diets (*P* < 0.001). In addition, significantly higher fecundity and male longevity levels were shown by the adult *Ae. aegypti* (*P* < 0.05) reared under diet D_2_. Based on the above, the larval diet D_2_ performed significantly better or equally to the IAEA diet, suggesting that the new formulation is acceptable in terms of nutrition and energetic components. Since the D_2_ diet is derived with readily available local ingredients that are relatively inexpensive, it can be recommended as an alternative diet for mass rearing of *Ae. aegypti* for area-wide IIT and SIT-based vector control in Sri Lanka.

## Figures and Tables

**Table 1:** Morphometric parameters and pupation success of *Ae. aegypti* 4^th^ instar larvae (mean ± SE) fed different larval diets.

Larval diet	Head length (mm)	Head width (mm)	Thoracic length (mm)	Thoracic width (mm)	Abdominal length (mm)	Abdominal width (mm)	Total length (mm)	Pupation success (%)
D_1_	0.51 ± 0.02^b^ (0.49-0.53)	0.54 ± 0.02^b^ (0.52-0.56)	0.81 ± 0.01^b^ (0.80-0.82)	0.94 ± 0.01^b^ (0.93-0.95)	3.37 ± 0.04^b^ (3.33-3.41)	0.65 ± 0.03^b^ (0.61-0.68)	4.71 ± 0.03^c^ (4.68-4.74)	78.0 ± 1.5^b^ (76.5-79.5)
D_2_	0.57 ± 0.01^a^ (0.56-0.58)	0.60 ± 0.01^a^ (0.59-0.61)	0.88 ± 0.01^a^ (0.87-0.89)	1.04 ± 0.01^a^ (1.03-1.05)	3.59 ± 0.01^a^ (3.58-3.60)	0.70 ± 0.01^a^ (0.69-0.71)	4.98 ± 0.04^a^ (4.94-5.02)	89.0 ± 2.3^a^ (86.7-91.3)
D_3_	0.56 ± 0.01^a^ (0.55-0.57)	0.59 ± 0.01^a^ (0.58-0.60)	0.87 ± 0.01^a^ (0.86-0.88)	1.02 ± 0.02^a^ (1.00-1.04)	3.50 ± 0.03^a^ (3.47-3.53)	0.67 ± 0.02^a,b^ (0.65-0.69)	4.85 ± 0.03^b^ (4.82-4.88)	83.6 ± 3.4^a^ (80.2-87.0)

Note: values are the mean ± Standard Error (SE) with the range in parenthesis. Different letters in a column denote significant differences (*P* < 0.05) at a 95% level of confidence based on the General Linear Model followed by Tukey's pairwise comparison.

**Table 2:** Morphometric parameters of *Ae. aegypti* adults fed different larval diets.

Larval diet	Wing length (mm)		Wing width (mm)		Thoracic length (mm)		Thoracic width (mm)		Abdominal length (mm)		Abdominal width (mm)	
	M	F	M	F	M	F	M	F	M	F	M	F
D_1_	5.09 ± 0.03^b^ (5.06-5.12)	5.10 ± 0.02^c^ (5.08-5.12)	1.06 ± 0.02^b^ (1.04-1.08)	1.32 ± 0.02^a,b^ (1.30-1.34)	1.65 ± 0.01^b^ (1.64-1.66)	1.82 ± 0.01^b^ (1.81-1.83)	1.21 ± 0.01^b^ (1.20-1.22)	1.38 ± 0.02^b^ (1.36-1.40)	4.05 ± 0.03^b^ (4.02-4.08)	4.08 ± 0.05^b^ (4.03-4.13)	0.68 ± 0.01^b^ (0.67-0.69)	0.86 ± 0.03^b^ (0.83-0.89)
D_2_	5.19 ± 0.03^a^ (5.16-5.22)	5.27 ± 0.04^a^ (5.23-5.31)	1.15 ± 0.02^a^ (1.13-1.17)	1.35 ± 0.02^a^ (1.33-1.37)	1.79 ± 0.01^a^ (1.78-1.80)	1.92 ± 0.02^a^ (1.90-1.94)	1.38 ± 0.02^a^ (1.36-1.40)	1.45 ± 0.02^a^ (1.43-1.47)	4.27 ± 0.04^a^ (4.23-4.31)	4.25 ± 0.05^a^ (4.20-4.30)	0.82 ± 0.04^a^ (0.78-0.86)	0.96 ± 0.03^a^ (0.93-0.99)
D_3_	5.11 ± 0.03^b^ (5.08-5.14)	5.19 ± 0.02^b^ (5.17-5.21)	1.14 ± 0.01^a^ (1.13-1.15)	1.30 ± 0.01^b^ (1.29-1.31)	1.78 ± 0.01^a^ (1.77-1.79)	1.88 ± 0.02^a^ (1.86-1.90)	1.35 ± 0.02^a^ (1.33-1.37)	1.43 ± 0.01^a,b^ (1.42-1.44)	4.18 ± 0.03^c^ (4.15-4.21)	4.19 ± 0.04^a^ (4.15-4.23)	0.70 ± 0.02^b^ (0.68-0.72)	0.96 ± 0.02^a^ (0.92-0.96)

Note: values are the mean ± Standard Error (SE) with the range in parenthesis. Different letters in a column denote significant differences (*P* < 0.05) at a 95% level of confidence based on the General Linear Model followed by Tukey's pairwise comparison.

**Table 3:** Life history parameters of *Ae. aegypti* fed different larval diets.

Larval diet	Fecundity (for 100 females)	Fertility (%)	Male survival time/longevity (days)	Flight ability (%)
D_1_	1364.2 ± 12.8^b^ (1351.4-1377.0)	95.4 ± 0.6^a^ (94.8-96.0)	15.5 ± 0.7^b^ (14.8-16.2)	96.0 ± 0.5^a^ (95.5-96.5)
D_2_	1453.4 ± 23.5^a^ (1429.9 ± 1476.9)	98.0 ± 0.3^a^ (97.7-98.3)	18.2 ± 0.4^a^ (17.8-18.6)	98.3 ± 0.2^a^ (98.1-98.5)
D_3_	1441.0 ± 20.1^a^ (1420.9-1461.1)	97.5 ± 0.5^a^ (97.0-98.0)	18.0 ± 0.3^a^ (17.7-18.3)	98.2 ± 0.2^a^ (98.0-98.4)

Note: values are the mean ± Standard Error (SE) with the range in parenthesis. Different letters in a column denote significant differences (*P* < 0.05) at a 95% level of confidence based on the General Linear Model followed by Tukey's pairwise comparison.

## Data Availability

The datasets supporting the conclusions of this article are included within the article.
